# Maternal adherence to the EAT-Lancet diet recommendations among pregnant women in Ireland and associations with offspring birth outcomes and childhood adiposity

**DOI:** 10.1007/s00394-025-03756-0

**Published:** 2025-07-15

**Authors:** Grégory Pen, Alexander Douglass, Celine M. Murrin, Cecily C. Kelleher, Adrien M. Aubert, Catherine M. Phillips

**Affiliations:** School of Public Health, Physiotherapy, and Sports Science, University College Dublin, Room F13, Woodview House, Belfield, Dublin 4, Ireland

**Keywords:** Dietary intake, EAT-Lancet commission, Lifeways, Pregnancy diet, Birth outcomes, Childhood obesity

## Abstract

**Background and aims:**

Pregnancy is a critical period during which maternal diet may impact offspring growth and future health. The EAT-Lancet commission promotes a plant-based diet pattern for meeting environmental and health challenges. EAT-Lancet diet scores have been developed however, thus far they have not been examined in the context of maternal-child health. We investigated adherence to EAT-Lancet diet recommendations during pregnancy and associations with offspring birth outcomes and childhood adiposity. The hypothesis tested was that greater adherence is not related to adverse outcomes.

**Methods:**

This study included mother–child pairs (n = 1,052) from the Lifeways Cross-generational cohort, in Ireland. Maternal EAT-Lancet diet score was derived from an early pregnancy validated food frequency questionnaire. Correlation analysis examined relationships with other diet scores (Healthy Eating Index (HEI-2015), Dietary Approach to Stop Hypertension (DASH) score, and energy adjusted-Dietary Inflammatory Index (E-DII). Logistic and linear regression analyses examined associations with offspring birth outcomes and childhood adiposity and weight status at age 5 and 9 years, with adjustment for energy intake and potential confounders.

**Results:**

The EAT-Lancet diet score (mean ± SD 21.0 ± 4.1) was moderately positively correlated with HEI-2015 and DASH scores (ρ > 0.5) and negatively correlated with dietary inflammation (E-DII (ρ < − 0.5, all *p*-value < 0.001)). Greater adherence to the EAT-Lancet diet recommendations was associated with higher intake of most micronutrients (except for retinol, riboflavin, vitamin B12 and calcium which were significantly lower). In energy-adjusted models, maternal EAT-Lancet diet score was positively associated with offspring birth weight (β 8.77, 95% CI 0.35–17.19, *p* = 0.04), length (β 0.06, 95% CI 0.01–0.1, *p* = 0.01), head circumference (β 0.04, 95% CI 0.01–0.07, *p* = 0.01) and lower likelihood of having a low birth weight (Odds ratio 0.91, 95% CI 0.85–0.99, *p* = 0.01). These associations did not withstand adjustment for additional confounders.

**Conclusions:**

Adherence to EAT-Lancet diet recommendations during pregnancy was not associated with adverse short or long-term offspring outcomes. Further examination in other cohorts with more diverse diets, and with additional health outcomes, is warranted.

**Supplementary Information:**

The online version contains supplementary material available at 10.1007/s00394-025-03756-0.

## Introduction

In 2019, the EAT-Lancet Commission on Food, Planet, Health tried to answer the question: “*Can we feed a future population of 10 billion people with a healthy diet within planetary boundaries?”.* The report arising from this work provides, for the first time, a definition of a planetary health diet and international recommendations [1]. Significant dietary changes are required to achieve improved health and environmental benefits, including a shift to a predominantly plant-based diet (PBD) rich in fruits, vegetables, nuts and legumes, by doubling their intake and a reduction by half of animal source food (particularly red meat) and sugar. A range of dietary scores reflecting the EAT-Lancet dietary recommendations have been developed, including the EAT-Lancet diet score [2, 3], the EAT-Lancet index [4], the Healthy Reference Diet score [5], the World Index for Sustainability and Health [6], or the Planetary Health Diet Index (PHDI) [7], and a growing body of evidence suggests that greater adherence to these recommendations is associated with a lower risk of mortality [4], diabetes and cardiovascular diseases [2, 5]. Moreover, greater adherence has also been linked with environmental benefits including lower carbon footprint and greenhouse gas emissions [2, 3, 7].

Although higher PHDI scores have also been linked with better overall dietary quality [7], the nutritional adequacy of PBDs, including the EAT-Lancet reference diet, has been questioned [8]. Nutritional deficiencies are of particular concern during pregnancy, which represents a critical developmental period for the foetus. According to the Developmental Origins of Health and Disease (DOHaD) paradigm [9], early-life exposures, such as maternal diet during pregnancy, may influence offspring development and their health trajectory. Adverse outcomes may include low birth weight (LBW), being small or large for gestational age (SGA and LGA, respectively), macrosomia, and childhood overweight and obesity (OWOB), which may track into adulthood [10], thus perpetuating an intergenerational disease-risk cycle.

A growing body of research, including our own, has investigated associations of maternal dietary scores with birth outcomes and childhood growth and adiposity [11–13]. These studies provide evidence that better diet quality and lower dietary inflammation are associated with both short-term (placental [14] and birth measures (weight and length), lower risk of being born LBW and SGA [15–17]), and long-term outcomes (bone measures, DNA methylation and lower risk of childhood OWOB) [18–20].

However, limited research on maternal adherence to EAT-Lancet diet recommendations has been conducted [21], and to our knowledge there are no studies which have examined this in the DOHaD context. Therefore, the aim of this work is to investigate the relationship between maternal adherence to EAT-Lancet diet recommendations during pregnancy and offspring birth outcomes and risk of being OWOB during childhood.

## Methods

### Study design and participants

As detailed in the cohort profiles [22, 23], the Lifeways cross-generational cohort study (Lifeways) is a prospective family cohort which aims to document health status, diet and lifestyle in the family members and to establish patterns and links across generations. Briefly, this longitudinal observational study was established between 2001 and 2003 with the recruitment of 1,124 expectant mothers during their first antenatal visit in two maternity hospitals in the Republic of Ireland. Multiple data were collected at baseline including a self-administered questionnaire on health status, diet and lifestyle. A sample of 1,094 live infants were born to these mothers (Supplemental Fig. 1), including 12 sets of twins (n = 1,082 mothers) excluded from these analyses due to differences in birth outcomes between singletons and twins, like BW. Follow-up of the cohort was conducted when children averaged ages 5 and 9 years. After exclusion of mothers with implausible energy intakes or insufficient dietary data, the current study included 1,052 mother–child pairs.

### Ethics

Ethical approval was granted by ethical committees of the Coombe University Hospital, Dublin, University College Dublin, Irish College of General Practitioners and University College Hospital, Galway, Ireland. Written informed consent was collected from all women upon recruitment and at all subsequent sweeps of the study.

### Dietary intake assessment

As already described elsewhere, dietary intake of the women during the first trimester of pregnancy was assessed using a validated semi-quantitative food frequency questionnaire (FFQ) of 149 items [22, 23]. Participants were asked about their average consumption frequency (9 levels, from ‘never or less than once per month’ to ‘6 + per day’) and a medium serving or common household unit was used as reference for each food item using standard portion sizes [24]. Daily quantities (grams/day) were derived by multiplying frequencies and portion sizes. The FFQ also assessed daily energy and crude nutrient intakes, that were computed using a program developed by the National Nutrition Surveillance Centre, School of Public Health, Physiotherapy and Sports Science, University College Dublin, Belfield, Dublin 4, Ireland and based on the McCance and Widdowson food tables [24]. Macronutrient intakes were also expressed as a percentage of energy intake using conversion factors of Mc Cance and Widdowson food tables. Calculation of micronutrient intake did not include micronutrient supplementation. Dietary scores including the HEI-2015 [25], the DASH score [26], and the E-DII [27, 28], were generated as previously described.

For this work, we excluded mothers who completed less than 20% of the FFQ (n = 5) or those with implausible energy intakes of less than 500 kcal or over 10,000 kcal (n = 7).

### Maternal EAT-lancet reference diet score generation

We assessed adherence to the EAT-Lancet reference diet based on a score described by Stubbendorff [4]. In addition to the original score (named “Original Stubbendorff EAT-Lancet score” and used in sensitivity analyses), we amended the score (named “Adapted Stubbendorff EAT-Lancet score”) to enhance alignment with the food components described in the EAT-Lancet recommendations. These modifications included: (i) merging “Beef and lamb” and “Pork” into a “Red meat” component, (ii) including a “Saturated fats” component and, (iii) converting dairy product content into milk equivalent to consider solid dairy foods into volume of milk needed to make it, according to the method described by Stockholm Resilience Centre, Stockholm University [29]. Supplemental Table 1 details the complete food item classification. Each food component received a score based on the proportion of adherence to the EAT-Lancet reference diet ranging from 0 to 3 depending on how this component is considered. For adequate component, a score of 3 was attributed for a consumption above the target intake (g/day) according to the EAT-Lancet reference diet and a score of 0 for a consumption < 50% of the target intake; whereas for moderate component, a score of 3 was attributed for a consumption below the target intake and a score of 0 for a consumption > 200% of the target intake. Both the original and adapted Stubbendorff EAT-Lancet scores ranged from 0 to 42 points. Table 1 details the complete scoring method (including intake ranges corresponding to values of 1 and 2).

**Table 1:** Criteria of deriving the original and adapted Stubbendorff EAT-Lancet scores (adapted from [4])

Food components	Target intake (g/day) according to EAT-Lancet reference diet	3 points	2 points	1 point	0 points	Details of scoring
**Adequate components**						
Vegetables	300 (200–600)	> 300	200–300	100–200	< 100	**Positive scoring (adequate component):** 3 points: Intake above target 2 points: lower limit of reference interval up to target 1 point: [50–100%[ of lower limit of reference interval 0 point: < 50% of lower limit of reference interval
Fruits	200 (100–300)	> 200	100–200	50–100	< 50	
Unsaturated fats	40 (20–80)	> 40	20–40	10–20	< 10	
Legumes	75 (0–150)	> 75	37.5–75	18.75–37.5	< 18.75	**Adjusted positive scoring (adequate component):**^a^ 3 points: Intake above target 2 points: [50–100%] of target 1 point: [25–50%[ of target 0 point: [0–25%[ of target intake
Nuts	50 (0–100)	> 50	25–50	12.5–25	< 12.5	
Whole grains	232	> 232	116–232	58–116	< 58	
Fish and seafoods	28 (0–100)	> 28	14–28	7–14	< 7	
**Moderate components**						
Red meat^b^	14 (0–28)	< 15	14–28	28–56	> 57	**Negative scoring (moderate component):** 3 points: Intake below target 2 points: Target intake to upper limit of reference interval 1 point:]100–200%] of upper limit of reference interval 0 point: > 200% of upper limit of reference interval
Beef and lamb	7 (0–14)	< 7	7–14	14–28	> 28	
Pork	7 (0–14)	< 7	7–14	14–28	> 28	
Chicken and poultry	29 (0–58)	< 29	29–58	58–116	> 116	
Eggs	13 (0–25)	< 13	13–25	25–50	> 50	
Dairy foods	250 (0–500)	< 250	250–500	500–1000	> 1000	
Potatoes	50 (0–100)	< 50	50–100	100–200	> 200	
Added sugars^c^	31 (0–31)	< 31	31–62	62–124	> 124	
Saturated fats^d^	11.8 (0–11.8)	< 11.8	11.8–23.6	23.6–47.2	> 47.2	

^**a**^Initial criteria for the positive score were not applicable for Legumes, Nuts, Whole grains and Fish and seafoods, as the lower limit of the reference interval was set to 0 for those foods

^**b**^The targets are divided by 2 for “Beef and lamb” and “Pork” for the original Stubbendorff EAT-Lancet score [4]

^**c**^Since the upper limits of the reference interval and target were identical, the upper reference interval of target intake was multiplied by 2 (62 g), that is in line with the WHO recommendation of ≤ 10% of the non-alcoholic energy intake [54]

^d^Added to the adapted Stubbendorff EAT-Lancet score but not in the original Stubbendorff EAT-Lancet score

### Offspring outcomes assessment

Birth measurements, including BW, length and head circumference, as well as infant sex and gestational age were collected from linked hospital records. From this information we derived different birth health outcomes: low birth weight (LBW) (BW < 2,500 g); macrosomia (BW > 4,000 g); SGA / adequate for gestational age (AGA) / LGA), indicating if the BW is less, appropriate, or more than expected for the gestational age, respectively, based on standard clinical cut-offs [30–32].

Childhood anthropometric measurements (height, weight, waist circumference) were taken at a follow-up home visit when the children were aged 5 by using a portable Leicester Height scale (Chasmors Ltd, London, UK), a SECA digital weighing scale, and a body tape with clear plastic slider (Chasmors Ltd, London), all following standardized protocols by trained research personnel, and by visiting their General Practitioner at 9 years old. The International Obesity Task Force (IOTF) references and cut-offs were used to determine body mass index (BMI) z-scores as well as to classify OWOB, taking child sex and age into account [33]. Waist circumference was used as a complementary measure of abdominal adiposity. In total, these outcomes were available for 561 and 274 children at 5 and 9 years of age, respectively.

### Confounders

The following variables, collected by self-completed questionnaires at baseline, are considered as potential confounders. Maternal sociodemographic characteristics included maternal age (years), educational level (below tertiary; ≥ tertiary) and parity. Additional measures of socioeconomic status, such as eligibility to the General Medical Services Scheme, a robust indicator of social disadvantage in Ireland [34], and weekly household income were included in descriptive tables only. Health and behavioural characteristics included pre-pregnancy BMI, smoking status and alcohol consumption during first trimester of pregnancy (current smokers/alcohol drinkers and women who have smoked/consumed alcohol during the last three months’ time prior to recruitment were classified as exposed); regular physical activity (physical activity such as jogging or cycling long enough to work up a sweat, at least once a week) during pregnancy and daily energy intake. Child sex and gestational age at birth were also considered as potential confounders for some outcomes.

### Statistical analysis

Diet scores, sociodemographic, health and behavioural characteristics and nutrient intakes were summarized overall and according to the quartiles of the adapted Stubbendorff EAT-Lancet score. Micronutrient intakes were compared to Irish (Health Service Executive [35]) recommendations or a harmonized set of Average Requirements [36] (based on EFSA [37] or Institute of Medicine recommendations [38]). Correlations between the different diet scores (original and adapted Stubbendorff EAT-Lancet, HEI-2015, DASH and E-DII) were examined using Spearman correlation analysis. Associations between maternal EAT-Lancet diet scores (original and adapted) and birth outcomes and childhood adiposity were investigated using linear (continuous outcome) and logistic (binary outcome) regressions. We estimated two models: (1) adjusted for energy intake, and (2) additionally adjusted for all other potential confounders. Child sex and gestational age were excluded from Model 2 for the following outcomes: SGA, LGA, BMI z-scores and OWOB, since they are considered in the calculation of these health outcomes. Sensitivity analyses were performed by replicating regression analyses i) after exclusion of mothers who provided less than 50% of answers or had an energy intake over 5,000 kcal (n = 38), and ii) using the original Stubbendorff EAT-Lancet score.

All analyses were carried using R Studio. Statistical significance was defined as a two-sided *p* value < 0.05.

## Results

### Maternal Stubbendorff EAT-Lancet scores and characteristics

Mothers had a mean ± SD adapted Stubbendorff EAT-Lancet score of 21.0 ± 4.1, age of 29.8 ± 5.9 years and BMI of 23.8 ± 4.2 (Table 2). Almost half of the mothers (49%) had at least a tertiary educational level, 26% had smoked and 69% had drank alcohol during the three months prior to recruitment. Examination of maternal characteristics according to quartiles of the adapted Stubbendorff EAT-Lancet score, revealed that mothers with higher scores, reflecting greater adherence, tended to have better quality (measured using HEI-2015 and DASH scores) and less pro-inflammatory (measured using E-DII score) diets, to be older, non-smokers, nulliparous and without medical card, and to have a higher education level, household income, regular physical activity and a lower daily energy intake (all *p* < 0.05).

**Table 2:** Sociodemographic and behavioural characteristics of the study participants overall and according to the adherence to the adapted EAT-Lancet diet score quartiles^a^

	All (n = 1052)	Quartile 1 (n = 292)	Quartile 2 (n = 292)	Quartile 3 (n = 257)	Quartile 4 (n = 211)	*p value*^b^
***Maternal sociodemographic characteristics***^*c*^						
Age at recruitment (years)	29.8 ± 5.9	27.1 ± 5.9	29.5 ± 6.2	30.9 ± 5.2	32.4 ± 4.6	< 0.001
Education level						< 0.001
Below tertiary	520 (51%)	173 (61%)	160 (57%)	120 (47%)	67 (32%)	
Tertiary or above	507 (49%)	110 (39%)	123 (43%)	134 (53%)	140 (68%)	
Household weekly income						0.003
≤ 600£	609 (65%)	181 (71%)	178 (68%)	142 (61%)	108 (56%)	
> 600£	335 (35%)	74 (29%)	84 (32%)	91 (39%)	86 (44%)	
Medical card (yes)	183 (18%)	66 (23%)	66 (23%)	31 (12%)	20 (10%)	< 0.001
Parity (multiparous)	573 (55%)	136 (47%)	171 (59%)	150 (59%)	116 (55%)	0.01
***Maternal health and behavioural characteristics***^c^						
Pre-pregnancy BMI (kg/m2)	23.8 ± 4.2	23.8 ± 4.4	24.1 ± 4.4	23.6 ± 4	23.7 ± 3.8	0.56
Smoking during first trimester of pregnancy (yes)	268 (26%)	107 (37%)	87 (30%)	46 (18%)	28 (14%)	< 0.001
Alcohol use during first trimester of pregnancy (yes)	720 (69%)	190 (66%)	201 (69%)	177 (69%)	152 (72%)	0.51
Regular physical activity (yes)	179 (19%)	35 (14%)	51 (19%)	46 (19%)	47 (26%)	0.03
Daily energy intake (kcal/day)	2529 ± 1037	2686 ± 1247	2553 ± 1029	2428 ± 900	2403 ± 840	0.006
***Maternal diet scores***						
Original Stubbendorff EAT-Lancet score	19.3 ± 4.3	14.5 ± 2.3	18.1 ± 1.5	21.1 ± 1.6	25.2 ± 2.8	< 0.001
Adapted Stubbendorff EAT-Lancet score	21.0 ± 4.1	16.0 ± 2.0	20.0 ± 0.8	22.9 ± 0.8	26.8 ± 2.0	< 0.001
HEI-2015 score	52.1 ± 8.4	46.0 ± 6.7	50.8 ± 7.3	54.8 ± 7.0	59.1 ± 7.0	< 0.001
DASH-score	23.7 ± 4.6	20.5 ± 3.6	22.5 ± 3.9	25.3 ± 3.4	27.9 ± 4	< 0.001
E-DII score	0.42 ± 1.77	1.63 ± 1.53	0.75 ± 1.52	-0.18 ± 1.42	-0.97 ± 1.47	< 0.001

^a^Values are means ± SD for continuous variables and n (%) for categorical variables (count and percentage of the category in the associated quartile)

^b^Kruskal–Wallis test for continuous variables or χ2 test for categorical variables.

^c^Missing information: mother age at recruitment (n = 2); education level (n = 25); household weekly income (n = 108); medical card (n = 8); parity (n = 10); prepregnancy BMI (n = 182); smoking status (n = 13); alcohol use (n = 4); regular physical activity (n = 120); energy intake (n = 0)

### Maternal Stubbendorff EAT-Lancet score and nutrient intakes

Mothers with higher adapted EAT-Lancet diet scores tended to have lower intake of total fat, SFAs and MUFAs (crude intake and %EI), cholesterol and total sugar (crude intake), and greater intake of PUFAs (crude intake and % EI), carbohydrates (crude intake), starch and fibre (crude intake and %EI) (Table 3). Regarding vitamins and micronutrients, they also tended to have higher intake of carotene, thiamine, niacin, folate, vitamins C, D, and E, iron, selenium, magnesium, copper and manganese, and lower intake of retinol, riboflavin, vitamin B12 and calcium (Table 4). Dietary recommendations for magnesium, riboflavin, vitamin B12 and calcium, were achieved across all adapted Stubbendorff EAT-Lancet score quartiles. Although mothers with a higher adherence had higher intake of vitamin D and E and iron,, dietary recommendations were insufficient for all quartiles. Mothers with the highest adapted Stubbendorff EAT-Lancet score (in the 4th quartile) were the only ones who met the recommendations for folate.

**Table 3:** Maternal macronutrient daily intakes during pregnancy according to maternal quartiles of adapted Stubbendorff EAT-Lancet score^a^

	All (n = 1052)	Quartile 1 (n = 292)	Quartile 2 (n = 292)	Quartile 3 (n = 257)	Quartile 4 (n = 211)	*p*-value^b^
Protein (g)	106.4 ± 51.0	110.8 ± 64.9	108.1 ± 44.7	102.5 ± 49.6	102.9 ± 36.9	0.31
Protein (% EI)^c^	17.0 ± 3.6	16.8 ± 4.3	17.2 ± 3.6	16.9 ± 3.1	17.4 ± 3.0	0.11
Fat (g)	101.8 ± 50.7	113.9 ± 60.9	105.2 ± 50.7	94.8 ± 43.0	89.2 ± 38.5	< 0.001
Fat (% EI)^c^	35.6 ± 6.4	37.6 ± 6.0	36.5 ± 6.4	34.6 ± 5.9	33.0 ± 6.1	< 0.001
MUFA (g)	32.6 ± 17.0	36.2 ± 19.7	33.7 ± 16.9	30.7 ± 15.9	28.6 ± 12.7	< 0.001
MUFA (% EI)^c^	11.43 ± 2.4	11.98 ± 2.2	11.73 ± 2.6	11.15 ± 2.5	10.60 ± 2.3	< 0.001
PUFA (g)	16.0 ± 9.5	15.2 ± 9.4	16.3 ± 11.0	16.3 ± 8.9	16.4 ± 7.8	0.01
PUFA (% EI)^c^	5.7 ± 2.1	5.0 ± 1.6	5.7 ± 2.3	6.0 ± 2.1	6.2 ± 2.0	< 0.001
SFA (g)	40.0 ± 21.9	48.3 ± 26.7	41.6 ± 21.1	35.5 ± 17.3	32.0 ± 15.7	< 0.001
SFA (% EI)^c^	13.9 ± 3.7	15.9 ± 3.7	14.4 ± 3.6	12.9 ± 3.2	11.7 ± 3.0	< 0.001
Cholesterol (mg)	329.2 ± 177.2	387.5 ± 227.1	338.4 ± 158.0	296.4 ± 156.2	275.8 ± 111.4	< 0.001
Carbohydrate (g)	314.4 ± 131.1	322.0 ± 152.7	311.9 ± 133.4	308.3 ± 113.0	314.8 ± 115.5	0.8
Carbohydrate (% EI)^c^	50.1 ± 7.4	48.2 ± 7.8	49.2 ± 7.6	51.4 ± 6.8	52.6 ± 6.4	< 0.001
Total sugar (g)	145.5 ± 70.9	158.5 ± 88.3	144.9 ± 67.2	139.6 ± 59.6	135.6 ± 58.0	0.04
Total sugar (% EI)^c^	21.7 ± 6.1	22.1 ± 6.9	21.5 ± 6.4	21.7 ± 5.7	21.2 ± 4.8	0.52
Starch (g)	166.4 ± 79.8	161.1 ± 85.3	164.6 ± 87.6	166.3 ± 71.6	176.4 ± 69.0	< 0.001
Starch (% EI)^c^	25.0 ± 6.2	22.8 ± 6.0	24.3 ± 6.2	26.0 ± 5.8	27.6 ± 5.4	< 0.001
Fibre (g)	27.1 ± 11.6	22.9 ± 10.0	26.0 ± 12.0	28.5 ± 11.4	32.6 ± 11.0	< 0.001
Fibre (% EI)^c^	2.2 ± 0.6	1.8 ± 0.5	2.1 ± 0.5	2.4 ± 0.5	2.8 ± 0.5	< 0.001

^a^Values are means ± SD

^b^Kruskall-Wallis test

^c^Energy intake proportion

**Table 4:** Maternal micronutrient daily intake during pregnancy according to maternal quartiles of the adapted Stubbendorff EAT-Lancet score and compared to reference recommendations^a^

	All participants (n = 1052)	Quartile 1 (n = 292)	Quartile 2 (n = 292)	Quartile 3 (n = 257)	Quartile 4 (n = 211)	*p* value^b^	Reference recommendation
Retinol (μg)	581.3 ± 684.8	707.4 ± 1101.6	588.2 ± 461.7	497.0 ± 362.3	499.8 ± 415.3	< 0.001	–
Retinol Equivalent(μg)	1018.7 ± 768.7	1055.7 ± 1157.9	1006.5 ± 606.3	970.3 ± 511.5	1043.3 ± 515.3	0.45	540^d^
Carotene (μg)	2868.4 ± 1741.4	2296.9 ± 1385.2	2715.7 ± 1953.0	3112.4 ± 1698.2	3573.7 ± 1630.4	< 0.001	–
Thiamine (mg)	2.1 ± 1.0	2.1 ± 1.2	2.1 ± 1.0	2.1 ± 0.8	2.3 ± 0.8	< 0.001	1.2^d^
Riboflavin (mg)	2.3 ± 1.2	2.5 ± 1.3	2.4 ± 1.2	2.1 ± 1.0	2.2 ± 1.1	< 0.001	1.5^d^
Niacin (mg)	25.4 ± 12.6	25.4 ± 16.4	25.0 ± 10.3	25.0 ± 12.1	26.5 ± 9.4	0.003	14^d^
Vitamin B6 (mg)	3.3 ± 1.3	3.3 ± 1.5	3.3 ± 1.4	3.2 ± 1.2	3.3 ± 1.0	0.2	1.5^d^
Vitamin B12 (μg)	5.7 ± 3.6	6.2 ± 4.4	6.1 ± 3.7	5.2 ± 2.9	5.1 ± 2.8	< 0.001	2.2^d^
Folate (μg)	368.6 ± 146.4	344.5 ± 151.1	359.3 ± 157.6	373.9 ± 126.8	408.5 ± 138.0	< 0.001	400^c^
Vitamin C (mg)	184.4 ± 107.9	147.2 ± 95.9	168.4 ± 105.7	209.2 ± 110.7	227.7 ± 101.0	< 0.001	80^d^
Vitamin D (μg)	3.6 ± 2.2	3.5 ± 2.4	3.4 ± 1.9	3.7 ± 2.4	3.8 ± 2.1	0.03	15^c^
Vitamin E (mg)	9.3 ± 4.8	8.4 ± 4.9	9.1 ± 5.3	9.7 ± 4.2	10.6 ± 4.6	< 0.001	12^d^
Phosphorous (mg)	1770.8 ± 765.5	1843.6 ± 839.8	1780.5 ± 749.3	1701.5 ± 721.4	1741.2 ± 727.2	0.25	580^d^
Calcium (mg)	1214.7 ± 691.5	1312.6 ± 734.9	1235.2 ± 694.0	1138.6 ± 626.0	1143.6 ± 688.0	0.02	1000^c^
Iron (mg)	13.2 ± 6.0	12.0 ± 6.0	12.8 ± 6.0	13.3 ± 5.7	15.3 ± 6.2	< 0.001	16^c^
Selenium (μg)	62.1 ± 31.3	59.6 ± 33.4	62.4 ± 33.7	62.2 ± 29.9	65.0 ± 26.3	0.01	49^d^
Zinc (mg)	12.0 ± 6.9	12.4 ± 9.0	12.5 ± 6.5	11.6 ± 6.3	11.5 ± 4.5	0.14	[7.5–11.5]^d^
Sodium (mg)	3296.0 ± 1569.3	3317.5 ± 1744.4	3333.5 ± 1586.6	3200.6 ± 1621.5	3330.6 ± 1183.2	0.22	2000^e^
Potassium (mg)	4247.6 ± 1577.9	4258.1 ± 1704.0	4248.5 ± 1642.2	4201.9 ± 1495.6	4287.2 ± 1403.1	0.83	3500^e^
Magnesium (mg)	352.5 ± 143.5	333.5 ± 132.1	349.2 ± 149.8	355.5 ± 144.8	379.6 ± 144.8	< 0.001	290–300^d^
Copper (mg)	1.3 ± 0.6	1.2 ± 0.6	1.3 ± 0.6	1.3 ± 0.6	1.4 ± 0.5	< 0.001	0.8^d^
Chloride (mg)	5271.1 ± 2663.0	5287.4 ± 2827.5	5372.4 ± 2780.8	5125.3 ± 2885.6	5286.1 ± 1872.3	0.23	3100^e^
Manganese (mg)	3.4 ± 1.6	2.9 ± 1.2	3.3 ± 1.6	3.6 ± 1.6	4.2 ± 1.8	< 0.001	2.4^e^
Iodine (μg)	216.9 ± 115.1	225.3 ± 113.5	224.1 ± 130.8	205.5 ± 95.1	209.0 ± 115.2	0.09	200^c^

^a^Values are means ± SD

^b^Kruskall-Wallis test

^c^Irish daily intake recommended for pregnant women [35]

^d^Harmonized values based on the European Food Safety Authority or the US Institute of Medicine Average Requirements for Pregnancy [36–38]

^e^ European Food Safety Authority daily recommendation, Average Intake [37]

### Correlation analyses

**Fig. 1:**
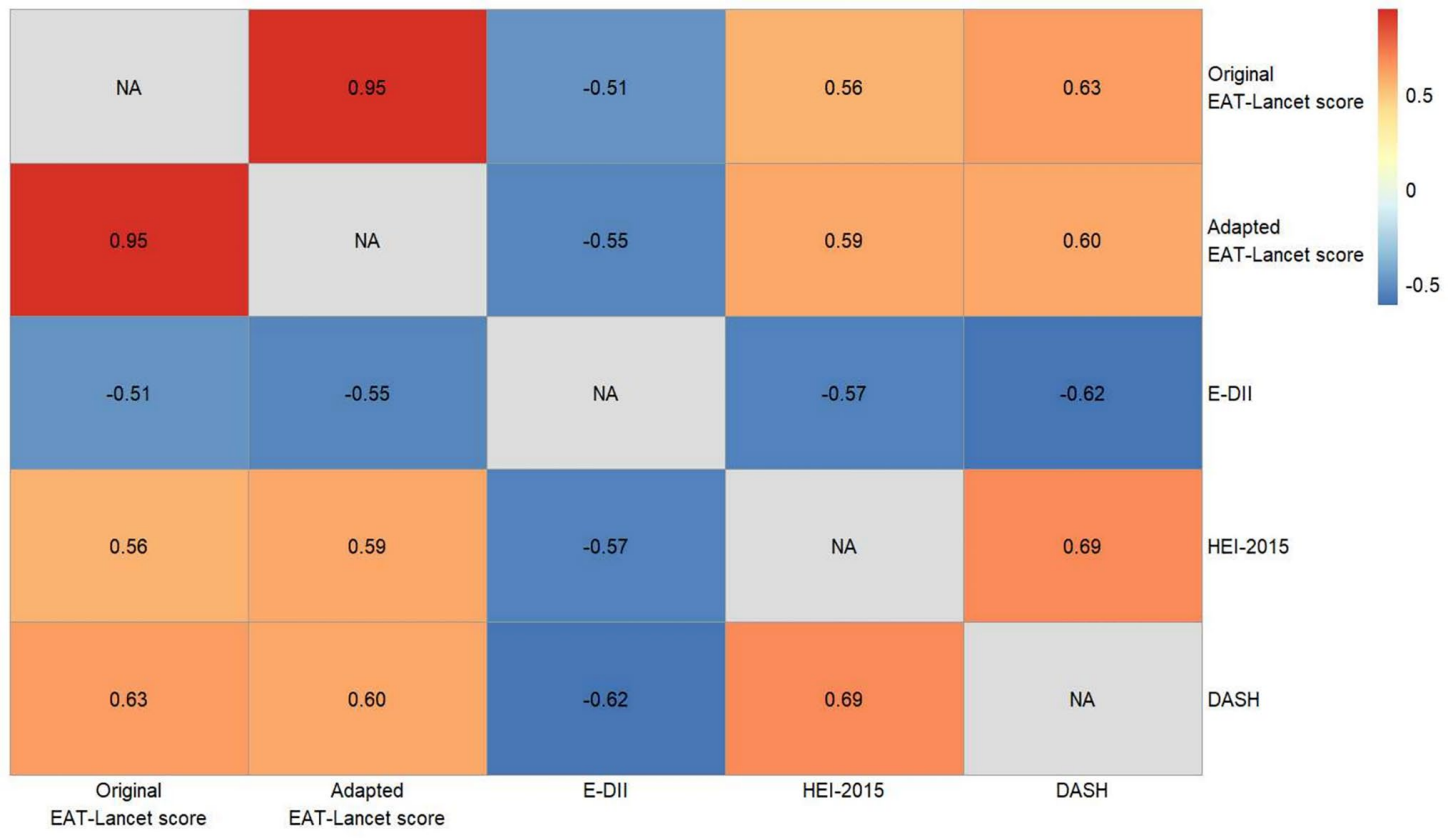
Heatmap of the Spearman’s correlation ρ-coefficients between the different diet 2 scores (all *p*-values <0.001). E-DII: Energy-adjusted dietary inflammatory index; DASH: 3 Dietary approaches to stop hypertension; HEI-2015: Healthy eating index-2015

The spearman correlations between the original and adapted versions of the Stubbendorff EAT-Lancet scores were very strong (ρ = 0.95, *p* < 0.001) (Fig. 1). The adapted Stubbendorff EAT-Lancet score was also positively correlated with the HEI-2015 score (ρ = 0.59, *p* < 0.001) and the DASH score (ρ = 0.60, *p* < 0.001) and negatively correlated with the E-DII score (ρ = − 0.55, *p* < 0.001).

### Associations between the maternal Stubbendorff EAT-Lancet score and birth outcomes and childhood adiposity

Offspring characteristics at birth and during childhood are presented overall and by quartiles of the adapted Stubbendorff EAT-Lancet score in Table 5. No significant association was observed between the adapted Stubbendorff EAT-Lancet score quartiles and the different offspring outcomes.

**Table 5:** Offspring health outcomes according to maternal quartiles of adapted Stubbendorff EAT-Lancet scores^a^

	All (n = 1052)	Quartile 1 (n = 292)	Quartile 2 (n = 292)	Quartile 3 (n = 257)	Quartile 4 (n = 211)	*p value*^b^
**Child birth outcomes**^*c*^						
Sex (Female)	532 (50.6%)	150 (51.3%)	144 (49.2%)	138 (53.6%)	100 (47.5%)	0.57
BW (g)	3515 ± 568	3472 ± 593	3469 ± 603	3598 ± 553	3536 ± 484	0.1
Birth length (cm)	50.7 ± 2.8	50.4 ± 2.9	50.6 ± 3.1	51.1 ± 2.7	50.9 ± 2.5	0.06
Low BW (yes)	42 (4.0%)	13 (4.5%)	17 (5.8%)	6 (2.3%)	6 (2.9%)	0.15
Macrosomia (yes)	183 (17.5%)	48 (16.5%)	43 (14.8%)	58 (22.6%)	34 (16.2%)	0.09
Head circumference (cm)	34.9 ± 1.8	34.8 ± 1.8	34.9 ± 2.2	35.0 ± 1.5	35.2 ± 1.4	0.11
Weight at GA						0.25
SGA	41 (3.9%)	12 (4.1%)	14 (4.8%)	8 (3.1%)	7 (3.3%)	
AGA	894 (85.0%)	252 (86.2%)	249 (85.2%)	209 (81.2%)	184 (87.1%)	
LGA	117 (11.1%)	28 (9.6%)	29 (9.9%)	40 (15.5%)	20 (9.5%)	
**Child anthropometric measurements during childhood**^*c*^						
*At 5 years*						
BMI						0.36
Normal	383 (69.6%)	93 (65.3%)	101 (73.0%)	100 (68.3%)	89 (71.6%)	
OWOB	151 (27.5%)	47 (33.0%)	34 (24.6%)	41 (28.0%)	29 (23.3%)	
Waist circumference (cm)	56.1 ± 4.5	56.3 ± 4.5	55.6 ± 4.2	56.3 ± 4.8	56.1 ± 4.6	0.54
*At 9 years*						
BMI						0.54
Normal	186 (66.9%)	40 (59.2%)	50 (74.0%)	50 (63.7%)	46 (69.1%)	
OWOB	74 (26.6%)	22 (32.6%)	12 (17.8%)	23 (29.3%)	17 (25.5%)	
Waist circumference (cm)	62.5 ± 10.7	64.3 ± 9.8	61. ± 12.4	61.9 ± 11.6	63.1 ± 8.4	0.82

^a^Values are means ± SD for continuous variables and n (%) for categorical variables (count and percentage of the category in the associated quartile)

^b^Kruskal–Wallis test for continuous variables or χ2 test for categorical variables. A bold *p-value* indicates that the test is significant (< 0.05)

^c^Missing information: sex (n = 1); BW (n = 4); length (n = 160); LBW (n = 4); macrosomia (n = 4); head circumference (n = 155); BMI category at 5 year follow-up (n = 518, whose 16 underweight cases) and 9 year follow-up (n = 791, whose 19 underweight cases); waist circumference at 5 year follow-up (n = 503) and 9 year follow-up (n = 774)

Linear and logistic regression analyses are presented in Table 6. In the energy-adjusted models the adapted Stubbendorff EAT-Lancet score was positively associated with BW (β: 8.77; 95% CI 0.35–17.19; *p* = 0.04), birth length (β 0.06; 95% CI 0.01–0.1; *p* = 0.01) and birth head circumference (β 0.04; 95% CI 0.01–0.07; *p* = 0.01). Offspring of mothers with greater adherence to the EAT-Lancet recommendations had approximately 10% lower likelihood of being born with a LBW (OR 0.91, 95% CI 0.85–0.99, *p* = 0.02). These associations were attenuated and did not remain significant in the fully adjusted model. No significant associations were observed with the other birth outcomes and with childhood OWOB at ages 5 and 9.

**Table 6:** Relationships between the adapted Stubbendorff EAT-Lancet score and the offspring health outcomes at birth, and 5 years old and 10 years old follow-up

	Model 1^d^			Model 2^d^		
	*β*^a^	95% CI	*p-value*^c^	*β*^a^	95% CI	*p-value*^c^
**Child birth outcomes**						
BW (g)	8.77	0.35, 17.19	**0.04**	-4.78	-16.56, 7.0	0.43
Length (cm)	0.06	0.01, 0.10	**0.01**	0.03	-0.05, 0.10	0.47
Head circumference (cm)	0.04	0.01, 0.07	**0.01**	-0.003	-0.04, 0.03	0.88

	Model 1^d^			Model 2^d^		
	Odds ratio^b^	95% CI	*p value*^c^	Odds ratio^b^	95% CI	*p value*^c^
LBW	0.91	0.85, 0.99	**0.02**	1.28	0.98, 1.75	0.09
Macrosomia	1.01	0.97, 1.05	0.65	0.98	0.90, 1.06	0.55
SGA^e^	0.98	0.90, 1.05	0.54	1.04	0.93, 1.17	0.45
LGA^e^	1.02	0.97, 1.07	0.42	0.97	0.92, 1.03	0.39

	Model 1^d^			Model 2^d^		
	*β*^a^	95% CI	*p value*^c^	*β*^a^	95% CI	*p value*^c^
**Child anthropometric measures**						
Waist circumference at 5y (cm)	0.01	− 0.03, 0.01	0.85	0.04	− 0.03, 0.08	0.69
BMI z-score at 5y (cm)^e^	− 0.01	− 0.08, 0.10	0.30	0.03	− 0.14, 0.21	0.33
Waist circumference at 9y (cm)^e^	− 0.07	− 0.08, 0.10	0.66	0.2	− 0.14, 0.21	0.42
BMI z-score at 9y^e^	0.01	− 0.40, 0.25	0.85	0.04	− 0.29, 0.70	0.69

	Model 1^d^			Model 2^d^		
	Odds ratio^b^	95% CI	*p value*^c^	Odds ratio^b^	95% CI	*p value*^c^
OWOB at 5y^e^	0.96	0.92, 1.01	0.13	0.95	0.90, 1.01	0.13
OWOB at 9y^e^	0.97	0.90, 1.03	0.32	0.98	0.89, 1.08	0.71

^a^Linear regression was applied to continuous growth outcomes

^b^Logistic regression was applied to binary health outcomes

^c^A bold *p-value* indicates that the test is significant (< 0.05)

^d^Model 1 was adjusted for energy intake. Model 2 was adjusted for energy intake, maternal age at recruitment, education level, parity, prepregnancy BMI, smoking status, alcohol status, physical activity, child sex and gestational age

^e^Health outcomes that take consider gestational age and child sex in their calculation were not adjusted for these 2 confounders in Model 2

Sensitivity analyses using the original Stubbendorff EAT-Lancet score and also using the adapted Stubbendorff EAT-Lancet score after exclusion of mothers who filled less than 50% of the FFQ or having an energy intake over 5,000 kcal, yielded similar results to those of the main analysis (Supplemental Tables 2 and 3).

## Discussion

In this study we adapted the EAT-Lancet diet score developed by Stubbendorff [4] to assess adherence to the EAT-Lancet diet recommendations among pregnant women and its associations with offspring birth outcomes and childhood adiposity up to age 9. Our score was moderately correlated with other diet scores in the expected directions (i.e., positively with diet quality and negatively with diet inflammatory potential). In regression models adjusted for maternal energy intake only, greater adherence to the adapted Stubbendorff EAT-Lancet reference diet was positively associated with birth measures (birth weight, length and head circumference), as well as a lower likelihood of having an infant with LBW. These results were robust to sensitivity analyses but did not remain significant after adjustment for other confounders. We did not observe any association with offspring adiposity and weight status during childhood.The EAT-Lancet reference diet is considered to be widely applicable to different populations, with positive health outcomes, such as reduced overall mortality [4, 5, 39, 40] and environmental issues, such as negative association with CO_2_ emissions [5, 39, 41]. However, there has been limited examination of the EAT-Lancet score in mothers [21] and to the best of our knowledge none during pregnancy or investigating intergenerational associations. The current novel work expands the knowledge base by examining the EAT-Lancet reference diet in the maternal-child context, addressing the DOHaD hypothesis. Mothers with a higher adherence to EAT-Lancet diet recommendations tended to have more favourable sociodemographic, health and behavioural characteristics, and diet of higher quality and lower inflammatory potential, consistent with previous works [15–17].

### Maternal plant-based diet during pregnancy and offspring health

We did not find any association between a greater adherence to the EAT-Lancet diet recommendations during pregnancy and adverse offspring outcomes at birth or during early or later childhood. Since the impact of the EAT-Lancet diet has not previously been investigated in the context of DOHaD, comparison with the literature is limited. Nevertheless, the EAT-Lancet reference diet is primarily a PBD and examination of PBDs in the context of DOHaD has generated conflicting findings. Data from the Danish National Birth Cohort (n = 66,738) showed no association of fish/poultry-vegetarian and lacto/ovo-vegetarian maternal diets with offspring BW and length [42]. A study from the USA highlighted that offspring of vegetarian (both fish/poultry-vegetarian and lacto/ovo-vegetarian) mothers were more likely to be SGA [43]. Whereas one study reported an association between a PBD pattern (derived by principal component analysis) during pregnancy and a higher risk of macrosomia in China [44].

### Maternal adherence to EAT-Lancet diet recommendations and macro- and micro-nutrient intakes

Regarding the nutrient intakes, in the current study mothers with higher adherence to the EAT-Lancet diet recommendations had higher intake of PUFAs, starch, fibre and most essential micronutrients and lower intake of total fat, SFAs, MUFAs, cholesterol, sugar and riboflavin, vitamin B12 and calcium. It is worth noting that intake of these micronutrients was above dietary recommendations. Conversely, although mothers with greater adherence to the EAT-Lancet diet recommendations had higher intake of vitamin D and E, and iron, none met dietary recommendations. This is not surprising given their low intake overall in this sample, particularly of Vitamin D which has been reported previously in Ireland, in which only 1.7% of mothers met recommendation during trimester 1 [45]. In addition, overall folate levels were below the recommended level and only mothers with the highest adherence (in the 4th quartile) met folate recommendations. Collectively these data highlight the importance of dietary improvements and/or supplementation for these key micronutrients during pregnancy [46]; deficiencies of which could negatively impact both the mother and offspring health or development. For instance, vitamin D deficiency during pregnancy has been associated with pregnancy complication, miscarriage, and greater risk of pre-term, SGA or LBW infants, some of which may be averted with vitamin D supplementation [47, 48].

### Strengths and limitations

Our study presents several strengths, although some limitations must also be noted. First, we adapted an existing score that is, according to the comparative review used for choosing the score, one of the best and most consistent scores for assessing adherence to the EAT Lancet recommendations [39]. These recommendations represent an important and timely topic of interest, not just for public health nutrition research but also for population and planetary health. Collectively, our results provide new insights, potentially opening up new avenues of research, and they contribute to building confidence in adopting EAT-Lancet diet recommendations during pregnancy. If replicated in other studies and populations these findings may have implications for clinicians, healthcare providers and dietary guidelines for pregnancy.

However, a limitation of the score (original and adapted) is that the EAT-Lancet recommendation values in grams/day do not consider caloric density or intake differences [7]. While we have controlled for maternal energy intake and several potential confounders, there may be other unmeasured confounders.

The Lifeways cohort is representative of a general obstetric population, however maternal birth outside Ireland was an exclusion criterion, which limits generalizability of the findings to other populations. Besides, it is also important to consider limitations of the EAT-Lancet recommendations in terms of equity, which is a focus of the second EAT-Lancet Commission [49]. Indeed, these recommendations need to take into account specific cultural and environmental contexts of populations [50]. For instance, it is particularly challenging for people with lower socioeconomic status [39], or populations presenting cultural or nutritional disparities [45], to meet these guidelines. Our population is a generally well-nourished high income population. Thus, results may only partly be generalizable to other populations and resource settings.

This study is strengthened by the longitudinal design, permitting examination of both short- and long-term offspring outcomes, and a relatively large sample size. Pregnancy diet was measured using a self-reported semi-quantitative FFQ, which is subject to information, recall and social desirability biases [51]. However, this FFQ has been validated and specifically designed for an Irish population. Additionally, results were robust to sensitivity analyses excluding mothers with implausible energy intake and those with insufficiently completed FFQs. Maternal diet was examined in the first trimester of pregnancy only, which is not necessarily representative of diet during the rest of pregnancy. However, previous research suggests that dietary intakes and patterns do not change substantially during pregnancy and are relatively stable [52, 53].

## Conclusions

There is growing interest in PBDs for meeting health and environmental issues. However, there are still concerns about potential risk of nutritional inadequacy. This study provides some new insights regarding maternal adherence to the EAT-Lancet diet recommendations during pregnancy and its associations with short and long-term offspring outcomes. Firstly, higher adherence to the EAT-Lancet diet recommendations was associated with higher intake of most micronutrients. Secondly, we did not find any evidence of an association with an adverse birth outcome or childhood OWOB. Further research is needed to investigate other child health outcomes and expand this investigation to other populations and paternal diet.

## Supplementary Information

Below is the link to the electronic supplementary material.

Supplementary file1 (DOCX 100 kb)

## Abbreviations

AGA: Adequate for gestational age
BW: Birth weight
CI: Confidence interval
DASH: Dietary approaches to stop hypertension
E-DII: Energy adjusted-dietary inflammatory index
FFQ: Food frequency questionnaire
HEI-2015: Healthy eating index
IOTF: International organisation task force
LBW: Low birth weight
LGA: Large for gestational age
OR: Odds ratio
OWOB: Overweight and obesity
PBDs: Plant-based diets
SGA: Small for gestational age
