# Supplementary material for: Maternal adherence to the EAT-Lancet diet recommendations among pregnant women in Ireland and associations with offspring birth outcomes and childhood adiposity
Source: Eur J Nutr. 2025 Jul 15;64(5):238. doi: 10.1007/s00394-025-03756-0 (PMC12263777; doi:10.1007/s00394-025-03756-0)
Supplement: Supplementary file 1 — Supplementary file1 (DOCX 100 kb) [file 394_2025_3756_MOESM1_ESM.docx]

**Supplementary material**

**
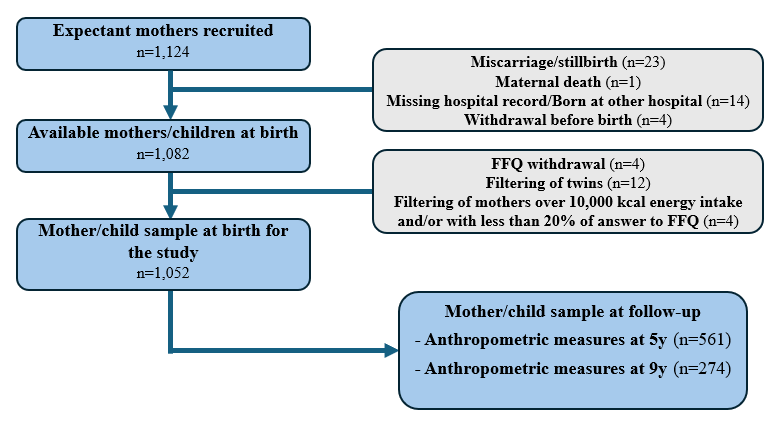
**

**Supplemental Figure 1.** Participant flow chart for the current analysis of the Lifeways study

**Supplemental Table 1.** Overall classification of food items from FFQ into the EAT-Lancet food components

| **Food components** | **Number of food items included** | **List of food items included (in order of appearance in the FFQ)** |
| --- | --- | --- |
| **Emphasized intake** |  |  |
| Vegetables | 23 | Carrots; Spinach; Broccoli, Spring Greens, Kale; Brussel Sprouts; Cabbage; Marrow, Courgettes; Cauliflower; Parsnips, Turnips; Leeks; Onions; Garlic; Mushrooms; Sweet peppers; Beansprouts; Green salad, Lettuce; Cucumber, Celery; Watercress; Tomatoes; Sweetcorn; Beetroot; Coleslaw; Avocado; Vegetable soups |
| Fruits | 11 | Apples; Pears; Oranges, Satsumas, Mandarins; Grapefruit; Bananas; Grapes; Melon; Peaches, Plums, Apricot; Strawberries, Raspberries, Kiwi Fruit; Tinned Fruit; Dried Fruit (e.g., raisins) |
| Added unsaturated fats | 4 | Sunflower margarine (e.g., Flora); Low-fat margarine (e.g., Low-low); Cream & Vegetable Oil spread (e.g., Golden Pasture, Kerry maid, Dairy Gold); Olive oil spread (e.g., Golden Olive) |
| Legumes | 5 (+1 from milk type) | Peas; Green Beans, Broad Beans, Runner Beans; Baked beans; Dried lentils, beans, peas; Tofu, Soya Meat, TVP, Vegeburger; Soya Milk |
| Nuts | 2 | Peanuts or other nuts; Peanut butter |
| Whole grains | 10 | Brown bread and rolls; Wholemeal bread and rolls; Crisp bread (e.g., Ryvita); Brown soda bread; Porridge, Readybrek; All bran, Weetabix, Shredded Wheat; Branflakes, Bran Buds; Muesli (e.g., Country Store, Alpen, sugar coated); Brown rice; Wholemeal pasta |
| Fish | 8 | Fish fried in batter, as in fish and chips; Fish fried in breadcrumbs; Oven baked/grilled fish (in breadcrumbs or batter); Fish fingers/fish cakes; Other white fish fresh or frozen (e.g., cod, haddock, plaice, sole, halibut, coli); Oily fish, fresh or canned (e.g., mackerel, kippers, tuna, salmon, sardines, herring); Shellfish (e.g., crab, prawns, mussels); Fish, roe, taramasalata |
| **Limited intake** |  |  |
| Beef and lamb | 10 | Beef: roast; Beef: steak; Beef: mince; Beef: stew; Beef burger; Lamb: roast; Lamb: chops; Lamb: stew; Corned beef, Spam, Luncheon meats; Liver, heart, kidney |
| Pork | 7 | Pork: roast; Pork: chops; Pork: slices/escalopes; Bacon; Ham; Sausages, Frankfurters; Savoury pies (e.g., meat pie, pork pie, steak & kidney pie, sausage rolls) |
| *Red meat combined* | *17* | *Combination of all 17 food items from "Beef and lamb" and "Pork"* |
| Poultry | 2 | Chicken portion or other poultry (e.g., turkey: roast); Liver paté |
| Eggs | 1 | Eggs as boiled, fried, scrambled, poached |
| Dairy | 8 (+6 from milk type) | Full-fat yoghurt or Greek-style yoghurt; Low fat yoghurt, fromage frais; Dairy desserts; Cheddar cheese; Brie, Edam type cheese; Low-fat cheddar cheese; Cottage cheese, cream cheese, low-fat soft cheese; Milk puddings (e.g., rice, custard, trifle); Full-fat milk; Low-fat milk; Skimmed milk; High-low milk; Buttermilk; Dried milk |
| Potatoes | 5 | Boiled, instant or jacket potatoes; Mashed potatoes; Chips; Roast potatoes; Potato salad |
| Added sugar | 6 | Ice cream, choc ices, Frozen desserts; Sweets, toffees, mints; Sugar added to tea, coffee, cereal; Jam, marmalade, honey, syrup; Fizzy Soft drinks (e.g., Cocoa Cola); Fruit squash |
| Added saturated fats | 3 | Cream; Butter; Lite Butter (e.g., Dawn Lite) |

| **Supplemental Table 2.** Sensitivity analyses of the relationships between the adapted Stubbendorff EAT-Lancet score and the offspring health outcomes at birth, and 5 years old and 10 years old follow-up after exclusion of mothers without sufficient or plausible FFQ data | | | | | | | | | | | | |
| --- | --- | --- | --- | --- | --- | --- | --- | --- | --- | --- | --- | --- |
| **Child birth health outcomes** | | | | | | | | | | | | |
|  | **Model 1^d^** | | | |  | **Model 2^d^** | | | | | |  |
|  | *β*^a^ | 95% CI | *p-value*^c^ | | |  | *β*^a^ | | 95% CI | *p-value*^c^ | |  |
| BW (g) | 8.76 | 0.23, 17.30 | 0.04 | | |  | -1.06 | | -13.04, 10.9 | 0.86 | |  |
| Length (cm) | 0.05 | 0.01, 0.10 | 0.03 | | |  | 0.04 | | -0.04, 0.12 | 0.31 | |  |
| Head circumference (cm) | 0.04 | 0.01, 0.07 | 0.01 |  | | | | 0.01 | -0.03, 0.05 | 0.57 | |  |
|  | Odds ratio^b^ | 95% CI | *p-value*^c^ | |  | Odds ratio^b^ | | | 95% CI | *p-value*^c^ | |  |
| LBW | 0.92 | 0.85, 0.99 | 0.03 | | |  | 1.25 | | 0.94, 1.74 | 0.14 | |  |
| Macrosomia | 1.01 | 0.97, 1.05 | 0.68 | | |  | 0.98 | | 0.90, 1.07 | 0.64 | |  |
| SGA^e^ | 0.98 | 0.90, 1.05 | 0.53 | | |  | 1.05 | | 0.94, 1.18 | 0.4 | |  |
| LGA^e^ | 1.02 | 0.97, 1.07 | 0.44 | | |  | 0.97 | | 0.91, 1.03 | 0.28 | |  |
| **Anthropometric health outcomes** | | | | | | | | | | | |  |
|  | **Model 1^d^** | | | |  | | | **Model 2^d^** | | | |  |
|  | *β*^a^ | 95% CI | *p-value*^c^ | |  | | *β*^a^ | | 95% CI | *p-value*^c^ | |  |
| Waist circumference at 5y (cm) | -0.004 | -0.04, 0.03 | 0.81 | |  | | -0.01 | | -0.06, 0.04 | 0.64 | |  |
| BMI z-score at 5y (cm)^e^ | -0.01 | -0.03, 0.01 | 0.45 | |  | | 0.03 | | -0.02, 0.08 | 0.27 | |  |
| Waist at 9y (cm)^e^ | -0.09 | -0.41, 0.24 | 0.61 | |  | | 0.24 | | -0.23, 0.70 | 0.32 | |  |
| BMI z-score at 9y^e^ | -0.004 | -0.04, 0.03 | 0.81 | |  | | -0.01 | | -0.06, 0.04 | 0.64 | |  |
|  | Odds ratio^b^ | 95% CI | *p-value*^c^ | |  | | Odds ratio^b^ | | 95% CI | *p-value*^c^ | |  |
| OWOB at 5y^e^ | 0.97 | 0.93, 1.02 | 0.22 | |  | | 0.96 | | 0.90, 1.02 | 0.22 | |  |
| OWOB at 9y^e^ | 0.97 | 0.90, 1.04 | 0.38 | |  | | 1.00 | | 0.91, 1.11 | 0.98 | |  |
| ^a^Linear regression was applied to continuous growth outcomes.  ^b^Logistic regression was applied to binary health outcomes.  ^c^A bold *p-value* indicates that the test is significant (<0.05).  ^d^Model 1 was adjusted for energy intake. Model 2 was adjusted for energy intake, maternal age at recruitment, education level, parity, prepregnancy BMI, smoking status, alcohol status, physical activity, child sex and gestational age  ^e^Health outcomes that take consider gestational age and child sex in their calculation were not adjusted for these 2 confounders in Model 2. | | | | | | | | | | |  |  |

| **Supplemental Table 3.** Sensitivity analyses of the relationships between the original Stubbendorff EAT-Lancet score and the offspring health outcomes at birth, and 5 years old and 10 years old follow-up | | | | | | | | | | | | | | |
| --- | --- | --- | --- | --- | --- | --- | --- | --- | --- | --- | --- | --- | --- | --- |
| ***Child birth health outcomes*** | | | | | | | | | | | | | | |
|  | | | **Model 1^d^** | | | | | |  | | **Model 2^d^** | | | |
|  | | | *β*^a^ | | 95% CI | | *p-value*^c^ | |  | | *β*^a^ | | 95% CI | *p-value*^c^ |
| BW (g) | | | 9.03 | | 1.10, 6.96 | | 0.03 | |  | | -.95 | | -12.14, 10.2 | 0.87 |
| Length (cm) | | | 0.05 | | 0.01, 0.09 | | 0.03 | |  | | 0.03 | | -0.04, 0.10 | 0.40 |
| Head circumference (cm) | | | 0.04 | | 0.01, 0.07 | | 0.003 | |  | | 0.00 | | -0.03, 0.04 | 0.88 |
|  | | | Odds ratio^b^ | | 95% CI | | *p-value*^c^ | |  | | Odds ratio^b^ | | 95% CI | *p-value*^c^ |
| LBW | | | 0.92 | | 0.85, 0.99 | | 0.02 | |  | | 1.20 | | 0.94, 1.61 | 0.17 |
| Macrosomia | | | 1.02 | | 0.98, 1.05 | | 0.41 | |  | | 1.00 | | 0.92, 1.08 | 0.95 |
| SGA^e^ | | | 0.97 | | 0.90, 1.04 | | 0.35 | |  | | 1.05 | | 0.94, 1.16 | 0.39 |
| LGA^e^ | | | 1.01 | | 0.97, 1.06 | | 0.67 | |  | | 0.97 | | 0.91, 1.03 | 0.27 |
| ***Child anthropometric measures*** | | | | | | | | | | | | | | |
|  | | | **Model 1^d^** | | | | | |  | | **Model 2^d^** | | | |
|  | | | *β*^a^ | | 95% CI | | *p-value*^c^ | |  | | *β*^a^ | | 95% CI | *p-value*^c^ |
| Waist circumference at 5y (cm) | | | 0.03 | | -0.06, 0.12 | | 0.46 | |  | | 0.07 | | -0.10, 0.24 | 0.44 |
| BMI z-score at 5y (cm)^e^ | | | -0.01 | | -0.03, 0.01 | | 0.48 | |  | | 0.03 | | -0.02, 0.08 | 0.25 |
| Waist circumference at 9y (cm)^e^ | | | -0.21 | | -0.53, 0.11 | | 0.19 | |  | | 0.05 | | -0.43, 0.52 | 0.85 |
| BMI z-score at 9y^e^ | | | -0.01 | | -0.04, 0.02 | | 0.62 | |  | | -0.01 | | -0.05, 0.04 | 0.70 |
|  | | | Odds ratio^b^ | | 95% CI | | *p-value*^c^ | |  | | Odds ratio^b^ | | 95% CI | *p-value*^c^ |
| OWOB at 5y^e^ | | | 0.99 | | 0.94, 1.03 | | 0.53 | |  | | 0.98 | | 0.92, 1.04 | 0.52 |
| OWOB at 9y^e^ | | | 0.95 | | 0.88, 1.02 | | 0.14 | |  | | 0.95 | | 0.86, 1.05 | 0.32 |
| ^a^Linear regression was applied to continuous growth outcomes.  ^b^Logistic regression was applied to binary health outcomes.  ^c^A bold *p-value* indicates that the test is significant (<0.05).  ^d^Model 1 was adjusted for energy intake. Model 2 was adjusted for energy intake, maternal age at recruitment, education level, parity, prepregnancy BMI, smoking status, alcohol status, physical activity, child sex and gestational age  ^e^Health outcomes that take consider gestational age and child sex in their calculation were not adjusted for these 2 confounders in Model 2. | | | | | | | | | | | | | | |
|  | |  |  | |  | |  | |  | |  | |  |  |
|  | |  |  | |  | |  | |  | |  | |  |  |
